# Application of Gaseous Ozone to Enhance Quality and Microbiological Safety of Strawberries

**DOI:** 10.3390/molecules31010042

**Published:** 2025-12-22

**Authors:** Miłosz Zardzewiały, Oskar Basara, Ireneusz Kapusta, Maciej Balawejder, Józef Gorzelany

**Affiliations:** 1 Department of Food and Agriculture Production Engineering, University of Rzeszow, 4 Zelwerowicza Street, 35-601 Rzeszów, Poland; 2 Department of Food Technology and Human Nutrition, University of Rzeszow, 4 Zelwerowicza Street, 35-601 Rzeszów, Poland; 3 Department of Chemistry and Food Toxicology, University of Rzeszow, 1 Ćwiklińskiej Street, 35-601 Rzeszów, Poland

**Keywords:** strawberries, gaseous ozone, physicochemical properties, microbial load

## Abstract

Ozonation represents one of the most promising non-thermal methods for enhancing the quality and storage safety of fresh fruits. In this study, the effects of gaseous ozone fumigation at different concentrations (10 and 50 ppm) and exposure times (10 and 20 min) on selected physicochemical and microbiological properties of strawberries during 7-day refrigerated storage were evaluated. Water content, mechanical properties, the profile and content of bioactive compounds (polyphenols, vitamin C), and antioxidant activity, as well as microbial counts and the dynamics of CO_2_ and ethylene production, were assessed. The results demonstrated that ozonation reduced water loss and slowed metabolic processes and fruit ripening, as indicated by lower CO_2_ and ethylene levels compared to the control. The application of ozone, particularly at the higher dose (50 ppm), contributed to maintaining higher vitamin C content and antioxidant activity and significantly reduced the number of mesophilic bacteria, yeasts, and molds, achieving reductions of approximately 1.86 log and 0.97 log on Day 7 compared with the untreated control, respectively. No adverse effects of ozonation on the mechanical properties of the fruit were observed. The findings confirm the relevance of gaseous ozone as a quality-enhancing elicitor and an effective tool for reducing microbiological contamination of fresh strawberries without compromising their properties.

## 1. Introduction

The strawberry (*Fragaria × ananassa* Duchesne) is one of the most popular fruits among consumers worldwide. *Fragaria × ananassa* Duchesne originated from a hybridization between the Chilean strawberry (*Fragaria chiloensis*) and the Virginia strawberry (*Fragaria virginiana*), carried out by Antoine Nicolas Duchesne in the 18th century. Strawberries belong to the Rosaceae family, which also includes economically important crops such as the apple (*Malus domestica*). They are cultivated in many countries and various climatic zones, but are most commonly grown in the temperate climate zone. Depending on the cultivation region, early, mid–early, mid–late and late cultivars can be distinguished, with selection influenced not only by weather, climate and environmental conditions, but also by agricultural tradition and available agrotechnical practices [1,2,3].

Global production of strawberries remains high: according to mapping data, the largest producers are China, the United States, Mexico, Egypt, Turkey, and Spain, while among European producers, high shares belong to Spain, Poland, Germany and the Netherlands [4]. Because strawberries are highly perishable, rapid postharvest cooling and short supply chains are essential. Typical commercial handling includes forced-air cooling shortly after harvest and cold storage to slow respiration and decay [5]. Furthermore, postharvest preservation worldwide involves various methods—modified-atmosphere packaging, sanitizing washes, and emerging non-thermal techniques such as gaseous ozone or UV-C treatments [6,7].

The health-promoting properties of strawberry fruits (*Fragaria × ananassa* Duchesne) result from their rich profile of bioactive compounds. Due to their high water content exceeding 90% and low caloric value of approximately 32 kcal/100 g, strawberries are recommended in weight-control diets and for obesity prevention [8]. The presence of polyphenolic compounds and vitamin C contributes to their antioxidant capacity. Phenolic compounds, including phenolic acids, exhibit a wide range of biological activities such as anticancer, anti-inflammatory, neuroprotective and antioxidant effects [9,10]. Strawberries are particularly rich in vitamin C, which is a potent antioxidant protecting the body against oxidative stress, supporting immune functions, reducing allergic responses and improving resistance to infections [11,12,13].

Strawberries are classified as non-climacteric fruits and must be harvested at full ripeness to achieve optimal sensory quality, particularly in terms of flavor, aroma and color. Key biochemical changes occur only during ripening while fruits remain attached to the plant [14]. Fruit softening during ripening is associated with thinning of cell walls, liquefaction of cell contents and loosening of intercellular bonds [15]. The presence of large cells and thin cell walls increases the susceptibility of strawberries to mechanical damage such as abrasions, cuts, bruises and juice leakage [16]. Mechanical damage is considered a form of stress occurring during harvest, transport and postharvest handling, leading to physiological and morphological changes that negatively affect fruit quality. Furthermore, biological and environmental stress factors contribute to the deterioration of harvested plant raw materials [17]. Mechanical injuries are among the most common and severe postharvest defects, causing substantial economic losses due to impaired appearance (skin and pulp browning, off-flavors) and induction of tissue breakdown [18]. The most commonly used parameter to assess fruit texture is firmness, which may be measured using puncture or compression tests by recording destructive force and deformation, depending on the analytical purpose [19]. Strawberry firmness depends on fruit size, ripeness stage, growing conditions and fruit load per plant [20]. Their tissue structure and high water content contribute to their particular sensitivity to damage [21].

Plant production depends primarily on soil–climate conditions and agrotechnical practices. High-quality yields require maintaining optimal cultivation conditions throughout the growing period [22]. A variety of biotic and abiotic factors can affect the quality of plant materials [23]. Among abiotic stress factors is ozone, described as an elicitor [24]. Ozonation has been shown to reduce water loss in stored plant products [25,26]. Controlled application of gaseous ozone induces defense responses in plant tissues, promoting increased synthesis of bioactive compounds [27]. Due to its strong oxidizing properties, ozone provides disinfection of plant products, thereby extending their shelf life [28]. Moreover, ozone activates resistance mechanisms against pathogens and functions as an abiotic elicitor, promoting defense-related metabolic responses in plant tissues [27]. Similar to other physical stimuli such as UV-C, ozone-induced elicitation involves oxidative and hormonal signaling pathways that underpin plant defense activation [29].

Mechanical injuries occurring during harvest and storage facilitate the penetration of pathogenic microorganisms into fruit tissues, leading to decay. Although fungicide application can reduce postharvest losses, fungal populations often develop resistance, which limits treatment effectiveness [30].

In commercial practice, several postharvest strategies are applied to delay deterioration of strawberries along the supply chain. Because fresh produce is highly susceptible to microbial contamination, sanitizing interventions remain an essential component of postharvest handling [31]. Besides rapid cooling, washing with chlorine-based sanitizing solutions (typically 50–200 mg/L free chlorine) is still widely used to reduce microbial loads on fresh fruits and vegetables [32]. Alternative oxidizing agents such as peracetic acid and hydrogen peroxide, as well as calcium chloride dips (0.5–6%), have also been tested to limit decay and maintain texture during storage [32]. More recently, gaseous or aqueous chlorine dioxide has been investigated specifically on strawberries, showing effective pathogen reduction and shelf-life extension [33,34]. All chemical treatments used in the strawberry industry are regulated, with maximum residue levels (MRLs) for active substances defined in the European Union under Regulation (EC) No 396/2005 [35] and additional limits for sanitizer residues established through EU and international food safety legislation.

Additionally, public concern regarding pesticide residues in food necessitates the development of alternative preservation methods [36]. Considering consumer safety and environmental protection, ozonation represents one of the most effective food preservation technologies already implemented in the food industry [37]. It is recognized as a non-thermal technique that does not generate environmental contamination and does not deteriorate product quality parameters [38].

In recent years, gaseous ozone has been increasingly investigated as a postharvest technology to improve the quality and microbiological safety of berries and, in particular, strawberries. Early work on small fruits demonstrated that ozone can inhibit fungal growth and slow pigment degradation in blackberry fruit during storage [39]. Later studies focused specifically on strawberries, showing that ozone generated in low-cost systems can help maintain fruit quality [40], that gaseous ozone is effective in inactivating foodborne pathogens and their surrogates on fresh and frozen strawberries [41], and that pre-packaging ozone treatments combined with modified atmosphere packaging (MAP) can influence the physicochemical, microbiological and sensory attributes of small berry fruits [42]. Other authors have reported that combining gaseous ozone with heat treatment can further extend the shelf life of fresh strawberries under cold storage [43], while comprehensive reviews have emphasized the potential of ozone to preserve quality and enhance microbial safety of fresh produce and have outlined remaining research needs [44]. More complex hurdle approaches, such as the combined use of aqueous ozone and ultrasound, have been shown to reduce microbial spoilage and pesticide residues while maintaining the storage quality of strawberries [45]. In addition, it has been demonstrated that strawberry cultivar markedly influences the effectiveness of postharvest gaseous ozone treatments on physicochemical, microbiological and bioactive properties of the fruit [46].

Despite this substantial body of work, there is still limited information on how relatively simple gaseous ozone fumigation regimes, using moderate concentrations and short exposure times, affect an integrated set of quality attributes (water status, texture, antioxidant-related parameters, ethylene production and microbial load) in specific dessert cultivars under realistic short-term refrigerated storage. In particular, little attention has been paid to widely grown Central European cultivars such as ‘Polka’, and to identifying ozone conditions that improve microbiological safety without compromising physicochemical quality. The present study addresses these gaps by evaluating two gaseous ozone concentrations (10 and 50 ppm) and two exposure times (10 and 20 min) applied to ‘Polka’ strawberries and systematically assessing their effects on selected physicochemical and microbiological properties during 7 days of cold storage.

Due to their high respiration rate, soft texture, and strong susceptibility to mechanical damage and microbial spoilage, strawberries are among the most perishable fruits in commercial distribution [47]. Under typical refrigerated conditions (0–4 °C), their shelf life rarely exceeds 5–7 days [5], especially when fruits are harvested at full ripeness [48]. In practice, this period includes postharvest cooling, transport, and distribution [49]. Therefore, applying methods that delay quality degradation—such as refrigeration, modified-atmosphere packaging (MAP), edible coatings, and non-thermal treatments—is essential [50]. In the present study, a 7-day refrigerated storage period was adopted as a realistic scenario for the distribution of dessert strawberries and to evaluate the effectiveness of gaseous ozone in maintaining their quality and microbiological safety.

The aim of the present study was to evaluate the effect of gaseous ozone fumigation at different concentrations on selected physicochemical and microbiological properties of strawberries during refrigerated storage.

## 2. Results and Discussion

### 2.1. Water Content in Strawberries During Storage

**Figure 1:**
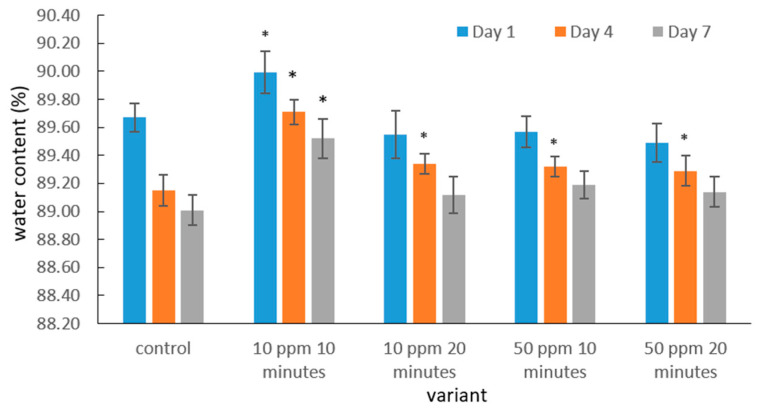
Water content in strawberry fruits in the control sample and after gaseous ozone fumigation during storage. Asterisks (*) indicate meaningful differences from the control within the same storage, *p* ≤ 0.05).

The water content of strawberry fruits [%] in the control sample and ozone-treated samples during storage is presented in Figure 1.

The water content of strawberry fruits varied depending on the storage duration and the applied gaseous ozone treatment. In all analyzed samples, including the control and ozone-treated fruits, the highest water content was recorded on the first day after ozonation, followed by a gradual decline throughout storage. This pattern is typical of soft fruits, which are characterized by a high transpiration rate driven by the vapor pressure deficit between the fruit surface and the surrounding air, as well as by intense postharvest metabolic activity. The latter contributes to structural weakening of tissues and accelerates moisture loss.

On the fourth day of storage, the water content decreased in all treatment variants; however, the decline was less pronounced in ozone-treated fruits than in the control. This suggests that moderate ozonation may temporarily slow down transpiration or help preserve cell integrity. Similar observations were reported by Zhang et al. [51], who indicated that ozone can modulate selected biochemical pathways and delay certain degradation processes in strawberries.

In contrast to the study by Zhang et al. [51], our experiment evaluated two simple, single-application doses of gaseous ozone rather than cyclic or aqueous ozonation protocols, and focused on the ‘Polka’ cultivar, which has not previously been characterized in this context. Moreover, we conducted a simultaneous analysis of a broader range of quality parameters, which allowed for a more comprehensive assessment of short-term postharvest responses to ozonation.

The highest water content on days 1, 4 and 7 was observed in fruits treated with 10 ppm ozone for 10 min, indicating that a mild ozone dose may support the maintenance of water balance in the early storage phase. This is consistent with findings from Piechowiak et al. [52], who demonstrated that low-dose ozone treatment, not inducing oxidative stress, can preserve membrane integrity and limit quality deterioration.

In contrast, fruits subjected to a higher ozone concentration (50 ppm) and longer exposure time (20 min) did not show significant differences in water content compared with the control on day 7. While this suggests that stronger ozonation does not enhance the preservation of water content under the tested conditions, the present dataset does not allow for a definitive conclusion regarding the overall efficacy of higher concentrations. Additional doses and longer fumigation times would be required to fully evaluate the potential benefits or limitations of more intensive ozone treatments.

### 2.2. Mechanical Properties of Strawberry Fruits

#### Puncture Force of the Skin and Flesh

**Figure 2:**
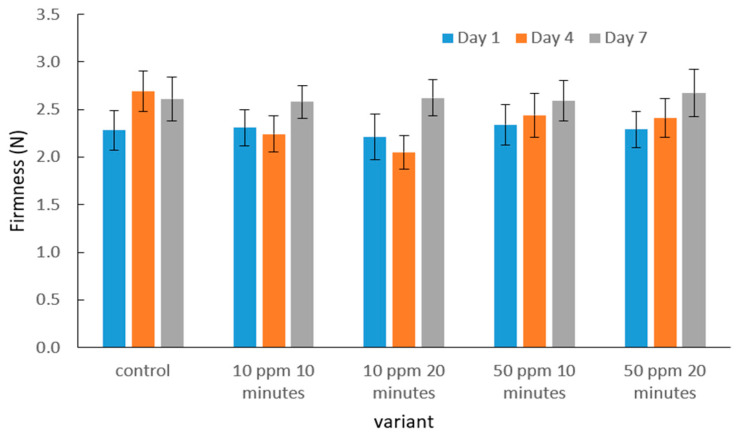
Effect of ozone dose and exposure time on the mean puncture force of the strawberry skin and flesh during storage. No significant differences were observed for firmness.

The mean destructive force values obtained during the puncture test of the strawberry skin and flesh after gaseous ozone treatment at different dose–time combinations, compared with the control sample, are presented in Figure 2.

Based on the obtained results, it can be observed that the destructive force values recorded during the puncture test of strawberry skin and flesh did not differ significantly between ozone-treated samples and the control. At all measurement points—after 1, 4, and 7 days of storage—the puncture force ranged from approximately 2.0 to 3.0 N. The lowest values for ozone-treated fruits were recorded on day 7, whereas the control samples showed the greatest decrease on day 4.

No clear relationship was noted between ozone dose, exposure time, and changes in puncture force—all treatment variants exhibited similar values, and the differences were statistically insignificant. Therefore, the applied ozone concentrations and exposure times did not significantly affect the mechanical resistance of strawberry skin and flesh, suggesting that ozone fumigation under the tested conditions did not alter this mechanical property.

The lack of statistically significant changes in textural characteristics following ozonation is supported by previous studies. Piechowiak et al. [52] reported that moderate ozone doses may even contribute to maintaining fruit firmness during storage by reducing surface microbial activity and limiting processes associated with cellular degradation. Similar observations were reported by Zhang et al. [51], who demonstrated that ozonated strawberries retain mechanical properties comparable to untreated fruits. Comparable behavior has also been documented for other fruit species. Basara et al. [53] showed that ozone treatment of quince fruit, under conditions similar to those applied in the present study, did not reduce tissue mechanical resistance and, in some cases, contributed to its stabilization. According to the authors, this effect may be associated with delayed cell wall degradation and inhibition of ripening-related processes.

Although the firmness of many strawberry cultivars typically decreases during storage, the control fruits in the present study did not show statistically significant softening over the 7-day period. This outcome may be associated with the relatively short storage duration and the use of refrigerated conditions, which noticeably slow down metabolic activity and cell wall degradation. Furthermore, the ‘Polka’ cultivar is known for its relatively stable texture during the early stages of postharvest storage, which may explain the limited softening observed in the control. These factors together likely contributed to the absence of significant differences between the control and ozone-treated samples.

### 2.3. Antioxidant Potential and Vitamin C Content

**Figure 3:**
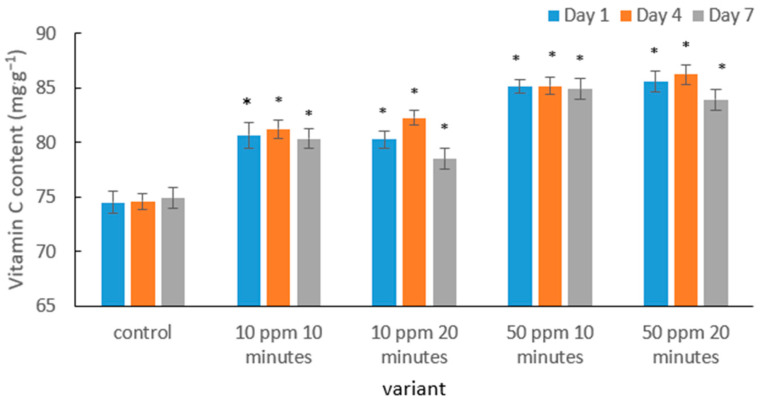
Mean vitamin C content in strawberry fruits in the control sample and after gaseous ozone treatment at different doses during storage. Asterisks (*) indicate meaningful differences from the control within the same storage, *p* ≤ 0.05).

The results indicate that ozone treatment had a clear effect on the vitamin C content in strawberry fruits, and the direction of these changes depended on the ozone dose and storage duration (Figure 3). In the control samples, ascorbic acid levels remained the lowest, which is consistent with the typical pattern of vitamin C degradation observed in soft fruits during storage. As reported in the literature, strawberries exhibit high metabolic activity after harvest, which accelerates vitamin C oxidation [54].

Ozone treatment at a dose of 10 ppm resulted in a noticeable increase in vitamin C content compared with the control. A similar protective effect was demonstrated in the study by Nurzakiyyah et al. [54], where immersion of strawberries in ozonated water (0.05 ppm) significantly improved ascorbic acid retention during storage. The authors attributed this effect to reduced surface microbial activity and a slowdown in oxidative processes within the fruit tissues.

A more pronounced effect was observed in fruits treated with a higher ozone concentration (50 ppm). In these variants, vitamin C content remained the highest regardless of exposure time. Comparable findings were reported by Zhang et al. [55], who showed that strawberries treated with gaseous ozone (4 ppm) exhibited slower vitamin C degradation. The authors suggested that moderate ozone doses may stabilize the antioxidant metabolism of fruits by reducing oxidative stress. This mechanism was also confirmed by Contigiani et al. [56], who demonstrated that washing strawberries in ozonated water not only inhibits pathogen development but also helps preserve antioxidant properties, including vitamin C concentration. Such stabilization of bioactive compounds results from the simultaneous reduction in enzymatic processes responsible for their degradation.

It is worth noting that the slight decrease in vitamin C content observed during storage in this study is consistent with findings by Pérez et al. [57], who reported that although ozonation delays vitamin C degradation, its gradual decline remains a natural consequence of ongoing metabolic activity. A broader mechanistic explanation was provided by Sachadyn-Król and Agriopoulou [27], who highlighted that ozone at low and moderate doses may act as a mild stressor activating enzymatic defense systems in fruit—including pathways responsible for regenerating ascorbic acid from oxidized forms. This may explain the temporary increase in vitamin C content observed in the 10 ppm/20 min treatment on day 4 of storage.

**Figure 4:**
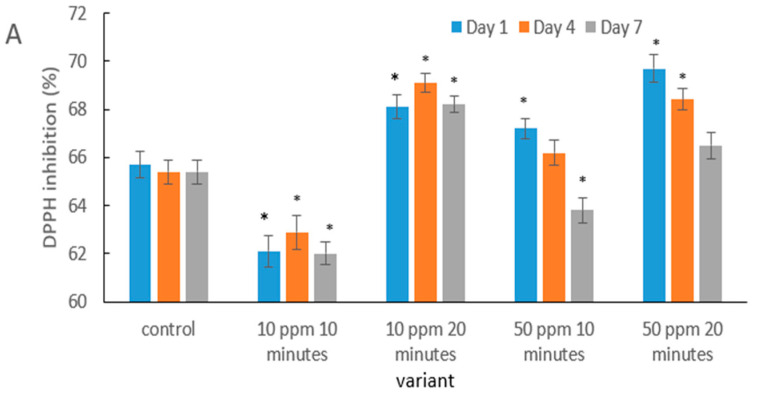

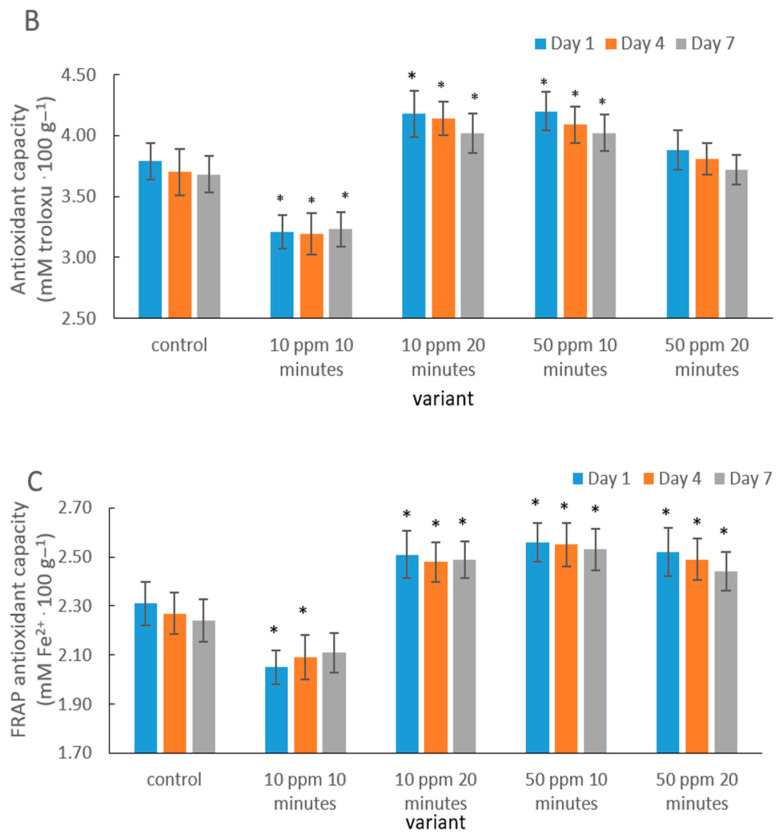
Antioxidant activity of strawberry fruits determined using the DPPH (**A**), ABTS (**B**), and FRAP (**C**) assays in the control sample and after gaseous ozone treatment at different doses during storage. Asterisks (*) indicate meaningful differences from the control within the same storage, *p* ≤ 0.05).

The obtained results indicate that the antioxidant response of strawberries to ozonation depends on both the concentration and the exposure time (Figure 4). A lower dose (10 ppm) combined with a short exposure could lead to an initial degradation of phenolic compounds and vitamin C, which was also observed in saskatoon berries after brief ozonation, as reported by Matłok et al. [58]. The authors confirmed that too short a contact with ozone may exert a pro-oxidative effect before cellular defense mechanisms are activated.

In contrast, a longer exposure at the same dose (10 ppm/20 min) enhanced DPPH (Figure 4A), ABTS and FRAP activity (Figure 4B,C). This effect may be interpreted as elicited oxidative stress that stimulates enzymes of the phenolic biosynthesis pathway. A similar phenomenon was documented in fresh-cut pitaya by Li et al. [59], where ozone treatment increased antioxidant activity through the stimulation of secondary metabolism.

The results obtained for the 50 ppm treatments (both 10 and 20 min) indicate that a higher ozone dose may exert a protective effect on bioactive compounds. A comparable mechanism was observed in citrus fruits: Iqbal et al. [60] reported increased antioxidant stability in Kinnow juice following ozonation, which was associated with the inhibition of enzymatic oxidation reactions.

Although a gradual reduction in antioxidant activity was observed over storage in most treatments, the rate of decline was slower in ozone-treated fruits. This aligns with Botondi et al. [38], who demonstrated that ozone can extend the stability of antioxidant bioactive compounds in berry fruits by reducing polyphenol and ascorbic acid degradation.

The more pronounced protective effect in the 10 ppm/20 min and 50 ppm (10 and 20 min) treatments supports the theory proposed by Sachadyn-Król and Agriopoulou [27], according to which, ozone, applied under properly optimized conditions, activates natural defense mechanisms and enhances the overall antioxidant capacity of fruit tissues.

### 2.4. Polyphenol Profile

Polyphenolic compounds in strawberries contribute to their antioxidant activity and health-promoting properties. Their content and composition may change as a result of ozone treatment and during storage. This subsection presents the results of the analysis of the polyphenol profile in strawberries subjected to different ozone treatment variants (Table 1).

The results indicate that the effect of ozonation on the total polyphenol content in strawberries is dose- and time-dependent. Initially (day 1), the highest values were recorded in the control sample and in the 50 ppm/20 min treatment, whereas fruits treated with the lower ozone dose (10 ppm) showed a clearly reduced polyphenol content. Such an initial decline in phenolic compounds following ozonation is consistent with observations in other plant materials, where easily oxidized phenolics (particularly anthocyanins) undergo degradation immediately after exposure due to the strong oxidizing action of ozone [51].

After four days of storage, a significant metabolic adjustment was observed—especially in the 10 ppm/10 min treatment, where the total polyphenol content exceeded that of the control. This type of delayed elicitation response has also been described for raspberries: moderate ozone doses resulted in an increase in phenolic levels and total antioxidant capacity during storage in comparison with untreated fruits [61]. It has been demonstrated that ozone can activate enzymes crucial for phenolic biosynthesis (e.g., PAL) and upregulate genes associated with the phenylpropanoid pathway, ultimately leading to a secondary accumulation of phenolic compounds in fruit tissues [62].

After seven days of storage, the highest total polyphenol content was found in strawberries treated with 10 ppm for 10 min—a result that strongly supports the concept of ozone as an abiotic elicitor. In a review by Sachadyn-Król and Agriopoulou [27], the authors emphasized that properly optimized low concentrations of ozone can enhance the accumulation of health-promoting secondary metabolites, including phenolics, in various plant products. Similar effects were reported for pitaya, where ozone treatment contributed to maintaining high phenolic and flavonoid levels, as well as improved antioxidant capacity [59].

In contrast, the lower polyphenol levels observed in fruits treated with higher ozone doses (50 ppm), particularly on day 4, may reflect excessive oxidative stress. High ozone concentrations or prolonged exposure times may lead to a dominance of degradation processes over elicitation, resulting in reduced phenolic content and decreased antioxidant activity [63]. This interpretation aligns with postharvest ozone processing reviews, which highlight the need for precise optimization of treatment conditions to avoid the degradation of sensitive bioactive compounds [64].

The phenolic profile obtained in this study—characterized by the dominance of pelargonidin glycosides and relatively greater stability of quercetin and kaempferol derivatives—is consistent with the known composition of strawberries, which are rich in pelargonidin-based anthocyanins and flavonols. It has been shown that anthocyanins are generally more susceptible to oxidative degradation than flavonols, which explains their stronger fluctuations during the initial days after ozonation, while quercetin and kaempferol may serve as an additional “antioxidant buffer” [55].

In summary, the most beneficial ozone treatment in relation to polyphenol retention in strawberries was the lower dose (10 ppm) combined with a short exposure time (10 min), which, over the course of storage, resulted in the highest total polyphenol content. In accordance with current scientific literature, ozone under such conditions not only limits the growth of surface microflora but may also function as a controlled stress factor, inducing defense mechanisms and phenolic biosynthesis in fruit tissues [27].

### 2.5. Microbial Load of Strawberry Fruits

Strawberries, due to their high water content and high water activity, provide a favorable environment for the development of microorganisms, including bacteria, yeasts and molds [65]. Their delicate structure, susceptibility to mechanical damage during harvesting, and lack of natural protective barriers promote rapid deterioration of microbiological quality. Microbial load is therefore a critical factor influencing both consumer safety and the storage stability of the product [66].

**Figure 5:**
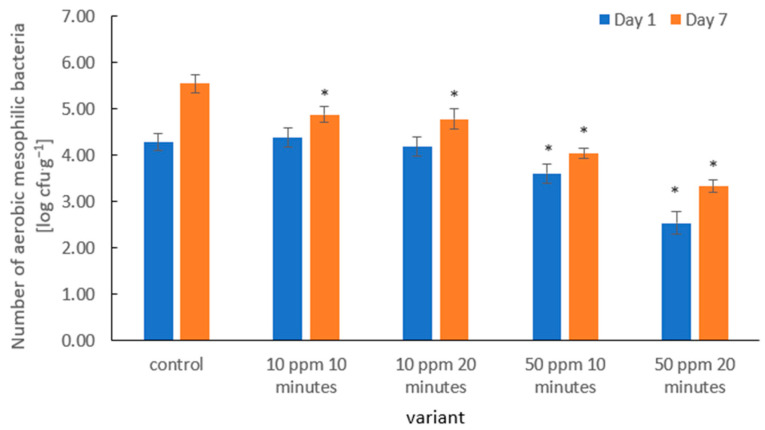
Aerobic mesophilic bacteria counts (log CFU·g^−1^) in strawberry fruits on Days 1 and 7 of refrigerated storage following ozone fumigation at different concentrations and exposure times. Bars represent mean values ± SD (*n* = 3). Asterisks (*) indicate meaningful differences from the control within the same storage, *p* ≤ 0.05).

The application of gaseous ozone in the tested variants effectively inhibited the growth of aerobic mesophilic bacteria on strawberry fruit during storage (Figure 5). In particular, the treatment with 50 ppm ozone for 20 min exhibited the strongest antimicrobial effect, indicating a clear dose-dependent relationship.

Previous studies confirmed that an increase in ozone concentration and longer exposure time can lead to stronger reductions in total mesophilic bacteria counts. Botondi [38] reported reductions of up to 2–3 log CFU·g^−1^ in fruits and vegetables following optimized gaseous ozone treatments. Moreover, Sarron et al. [67] demonstrated that even lower ozone doses (a few ppm) could reduce microbial counts by approximately 1 log CFU·g^−1^ in carrots and other vegetables, indicating that even moderate levels of ozone can significantly impact microbial populations.

The observed differences in antimicrobial efficacy between the 10 ppm and 50 ppm treatments in our study can be explained by the mechanism of ozone action: higher concentrations generate larger quantities of reactive oxygen species (ROS), which oxidize membrane lipids, proteins, and nucleic acids, resulting in faster microbial inactivation [68]. It has also been shown that prolonged exposure does not always proportionally increase microbial reduction. Under conditions of high organic matter or moisture levels, a portion of ozone is consumed in side reactions, reducing its bioavailability for direct antimicrobial action [38].

Importantly, the ozone treatments not only reduced the initial microbial counts but also slowed microbial proliferation during storage. The 50 ppm treatments resulted in the lowest bacterial levels on day 1 and maintained the slowest growth kinetics up to day 7. This suggests that higher ozone doses ensure more stable microbial suppression over time, not only an immediate reduction. Similar findings were reported by Gorzelany et al. [63], who observed that ozone treatment at 10 ppm for 15–30 min in Saskatoon berries effectively delayed the growth of aerobic mesophilic bacteria during storage.

**Figure 6:**
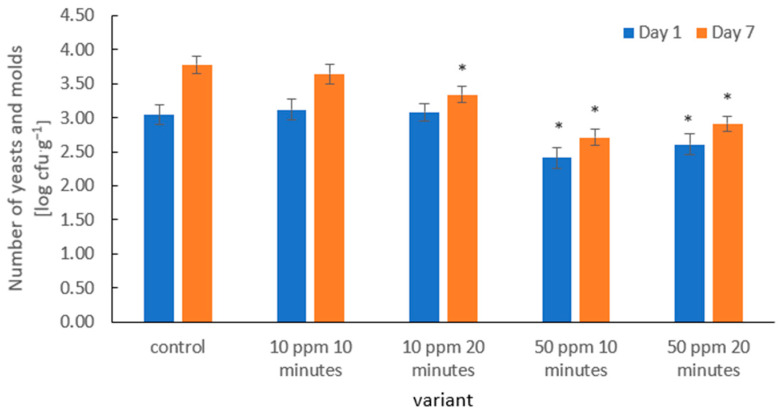
Yeast and mold counts (log CFU·g^−1^) in strawberries on days 1 and 7 of storage after ozone treatment. Error bars represent SD (*n* = 3). Asterisks (*) indicate meaningful differences from the control within the same storage, *p* ≤ 0.05).

In the presented results, the number of yeasts and molds in the control samples increased markedly during storage—from 3.04 log CFU·g^−1^ on day 1 to 3.78 log CFU·g^−1^ on day 7 (Figure 6). This behavior is characteristic of strawberries, which, due to their high water activity, delicate skin, and abundance of sugars, provide a suitable environment for fungal growth, as demonstrated by Ladika et al. [66].

Ozone treatment at 10 ppm (10 and 20 min) led to a moderate reduction in yeast and mold counts, particularly noticeable on day 7 of storage. This is consistent with the observations of Wu et al. [69], who reported that fumigation of fruits with ozone (various doses and exposure times) can significantly inhibit mold proliferation in a dose- and time-dependent manner.

The strongest antimicrobial effect was observed at an ozone concentration of 50 ppm. An exposure of 10 min reduced yeast and mold counts to 2.41 log CFU·g^−1^ on day 1, while 50 ppm for 20 min resulted in counts of 2.61 log CFU·g^−1^. This distinct dose–response relationship aligns with findings indicating that ozone exhibits strong fungicidal activity, particularly against mold species [70].

Importantly, the 50 ppm treatments maintained reduced microbial levels throughout storage, likely because fungal populations were sufficiently lowered or damaged, delaying their regrowth rather than providing a prolonged inhibitory effect of ozone. A similar sustained antifungal effect following ozone exposure was reported by Xue et al. [71].

The antimicrobial mechanism of ozone involves both its strong oxidative properties and the generation of reactive oxygen species (ROS), leading to membrane disruption, damage to fungal cell walls, and leakage of cellular contents. Studies on fungal conidia have shown that ozone exposure results in surface shrinkage, membrane deformation, and cytoplasmic disintegration—confirming oxidative and structural damage caused by ROS [38]. These results are consistent with the broader literature identifying ozone as one of the most effective non-thermal technologies for controlling fungal contamination in fresh fruits.

### 2.6. Changes in Carbon Dioxide Concentrations and Ethylene Production During Storage of Strawberry Fruit

**Figure 7:**
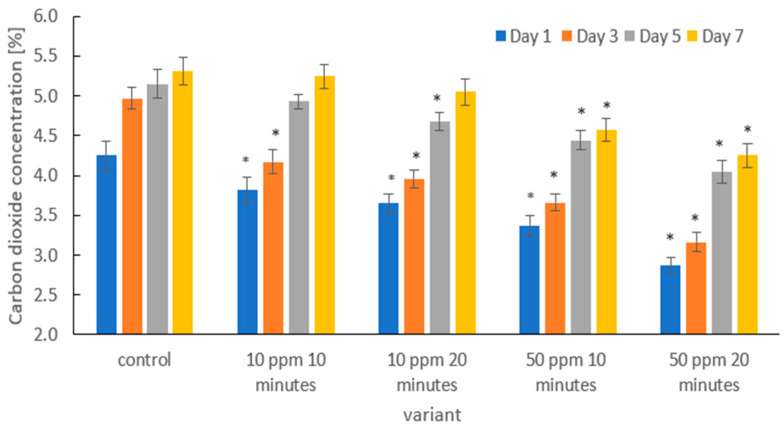
Changes in CO_2_ concentration [%] on days 1, 3, 5, and 7 of storage in strawberries subjected to different experimental treatments. Asterisks (*) indicate meaningful differences from the control within the same storage, *p* ≤ 0.05).

Figure 7 presents the changes in carbon dioxide (CO_2_) concentration inside the packaging containing strawberries subjected to ozone treatment at different dose–time combinations, compared with the untreated control. CO_2_ production was monitored on days 1, 3, 5, and 7 of refrigerated storage.

At all measurement points, the control samples exhibited the highest CO_2_ concentration, indicating the most intensive metabolic processes. Ozone treatment significantly reduced CO_2_ production compared with the control, and the magnitude of this effect was dose-dependent. The lowest impact was observed for the 10 ppm/10 min treatment, whereas the strongest reduction in CO_2_ concentration occurred in the 50 ppm/20 min variant.

A progressive increase in CO_2_ concentration was observed during storage in all treatments, reflecting intensifying respiration and ripening. However, the scale of this increase was markedly limited in ozone-treated fruits, indicating that ozone effectively slowed the dynamics of metabolic transformations. In particular, both 50 ppm treatments resulted in significantly lower CO_2_ concentrations from day 1 to day 7 compared with the control, with the lowest levels recorded in the 50 ppm/20 min treatment. These findings clearly confirm that ozone application immediately after harvest extends storage life by slowing down postharvest metabolism.

The obtained results are consistent with the study by Zhang et al. [55], who demonstrated that gaseous ozone treatment (4 ppm) significantly reduced fruit transpiration and delayed senescence in strawberries, thus prolonging shelf life. Piechowiak et al. [52] also reported beneficial effects of cyclic ozone treatments on the energy status of strawberry tissues (higher ATP levels and energy charge index) and improved mitochondrial enzyme activity, which was associated with delayed texture deterioration. Reduced transpiration rates following ozone application were also reported in papaya (*Carica papaya* L.) [72], melon [73], and coriander [74] compared with untreated controls.

**Figure 8:**
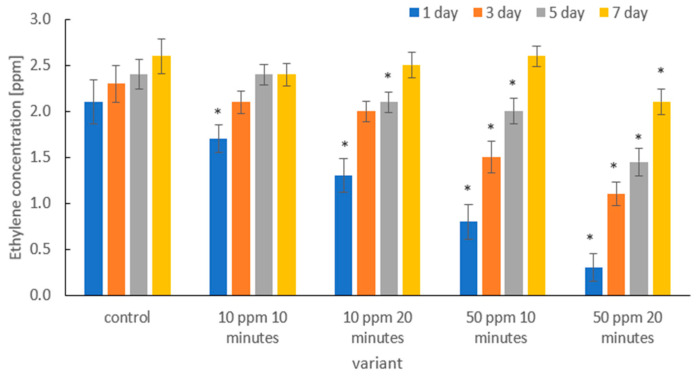
Changes in ethylene concentration [ppm] on days 1, 3, 5, and 7 of storage in strawberries subjected to different experimental ozone treatments. Asterisks (*) indicate meaningful differences from the control within the same storage, *p* ≤ 0.05).

Figure 8 shows the effect of gaseous ozone on ethylene concentration (ppm) in the headspace of packages containing strawberries on days 1, 3, 5, and 7 of storage, compared with the untreated control. Significant differences were observed among treatment variants, as well as between sampling days.

In the control samples, the highest ethylene concentration was recorded on day 1, and the levels remained relatively stable throughout storage, indicating continued metabolic activity and ripening processes. In contrast, ozone-treated samples showed reduced ethylene production, with the extent of reduction depending on ozone dose and exposure duration.

The treatment of 10 ppm for 15 min showed a decreased ethylene level on days 1 and 3, although the concentrations returned close to the control values on days 5 and 7. A similar pattern was observed for the 10 ppm/30 min treatment, where the initial reduction in ethylene was followed by a gradual increase during storage.

The strongest suppressive effect on ethylene biosynthesis was observed in fruit treated with ozone at 50 ppm for 30 min, where the ethylene concentration recorded on day 1 was the lowest in the entire experiment. Although ethylene in this variant showed a progressive increase over time, the values remained significantly lower than in the control throughout the storage period. The 50 ppm/15 min treatment showed an intermediate trend—a clear reduction in the early stage followed by a noticeable increase by day 7. Overall, the results demonstrate that higher ozone doses combined with longer exposure times are more effective in reducing ethylene production, although the efficiency of inhibition weakens with prolonged storage. This suggests that ozone treatment slows down enzymatic pathways related to ethylene biosynthesis, contributing to delayed senescence immediately after treatment, but the protective effect is gradually diminished as storage time increases.

Gaseous ozone inhibits ethylene production, thereby extending the storage life of fresh plant commodities [75]. Properly selected ozone concentrations can help maintain a low ethylene production rate during storage [76]. Elevated ethylene levels in storage chambers may accelerate fruit ripening and lead to premature deterioration. Therefore, the regulation and continuous control of ethylene concentration are crucial to ensure prolonged shelf life and reduced postharvest losses of horticultural products [77].

Several physiological mechanisms may explain the observed suppression of ethylene production in ozone-treated strawberries. Ozone can reduce the activity of ACC synthase and ACC oxidase, which are key enzymes in the ethylene biosynthetic pathway [78]. The temporary inhibition of these enzymes limits the conversion of S-adenosylmethionine to ACC and subsequently to ethylene. In addition, moderate ozone exposure may lower respiratory intensity and modify reactive oxygen species (ROS) signaling, leading to enhanced antioxidant enzyme activity and reduced oxidative stress [79], which are both associated with slower senescence. Ozone can also help to stabilize cell membranes by limiting lipid peroxidation [80], thereby delaying cell wall degradation and other ripening-related processes that stimulate ethylene synthesis. Previous studies confirm that ozone treatments can suppress ethylene generation and delay physiological aging in fruits during storage [81]. These combined effects suggest that ozone modulates both biochemical and structural pathways regulating ethylene production, resulting in a transient but measurable delay in ripening.

### 2.7. Regulatory and Practical Considerations for the Commercial Application of Ozone

When considering the potential commercial implementation of gaseous ozone as a postharvest technology, regulatory constraints and safety requirements must be taken into account. In the European Union (EU), ozone generated from oxygen is regulated under the Biocidal Products Regulation (BPR, Regulation (EU) No 528/2012), where it is classified as an active substance for product types including food and feed area disinfection. The most recently implemented regulation confirms its approval and specifies conditions for safe use in food environments: EU approval of ozone generated from oxygen [82], general European Chemicals Agency (ECHA) information on ozone as a biocidal substance [83].

Despite its decomposition to oxygen and absence of chemical residues on treated produce, ozone remains a strong oxidizing biocide and must be applied under controlled conditions to protect workers. Occupational exposure limits underscore these safety requirements. According to the Occupational Safety and Health Administration (OSHA) and the National Institute for Occupational Safety and Health (NIOSH), the permissible exposure limit (PEL) is 0.1 ppm as an 8 h time-weighted average (TWA), and the ceiling value recommended by NIOSH is also 0.1 ppm [84,85].

Scientific reviews consistently emphasize that while ozone is highly effective in microbial inactivation and can extend the shelf life of fresh produce, its use requires accurate concentration monitoring, controlled gas distribution, and safe engineering designs to avoid worker exposure [86]. A recent comprehensive review highlights both the benefits and limitations of ozone in postharvest handling, including challenges associated with dose–response optimization, ozone stability, and possible effects on sensory attributes depending on treatment intensity [87]. Regarding sensory quality, several studies indicate that properly controlled ozone treatments can reduce fungal decay while maintaining key fruit attributes such as aroma, firmness, and color. However, outcomes depend on dose and commodity type [88].

Because the present study did not assess potential ozone by-products nor measure worker exposure levels, further research is needed before scaling this technology to commercial facilities. Future work should include gas dispersion modeling in larger storage rooms, evaluation of occupational safety systems, and assessment of sensory characteristics of ozone-treated strawberries to ensure consumer acceptance.

## 3. Materials and Methods

### 3.1. Research Material

In a laboratory experiment, fruits of the strawberry cultivar ‘Polka’, originating from field cultivation, were used. For many years, this cultivar has been among the most highly valued dessert varieties in Poland. Its fruits are characterized by large size, high firmness, and an attractive, uniformly dark red skin color. The flesh is juicy, aromatic, and sweet, making ‘Polka’ well suited both for the fresh fruit market and for processing. Plants of this cultivar can be grown both in the field and under covers, and they are known for their high yield potential and good tolerance to environmental stress.

Despite its good tolerance under field conditions, the ‘Polka’ cultivar exhibits typical postharvest limitations characteristic of strawberries, such as susceptibility to loss of firmness, dehydration, and microbiological spoilage. For this reason, it is widely used in studies on postharvest physiology and storage methods, which makes it an appropriate model for evaluating the effectiveness of ozone treatment. The fruits used in the study were harvested manually in the second week of June 2025.

Immediately after harvest, the fruits were placed in clean, ventilated plastic trays and transported directly from the farm to the laboratory. The transport distance did not exceed 20 km, and the total time between harvest and arrival at the laboratory was approximately 1 h. During transport, the strawberries were kept under shaded conditions at ambient temperature but protected from direct sunlight and mechanical damage. Upon arrival at the laboratory, the fruits were immediately transferred to a cold room and stored at 2 ± 1 °C and relative humidity of approximately 90% until analysis. The pre-experimental storage period did not exceed 2 h, ensuring minimal physiological changes before the application of ozone treatments.

Before the experiment began, the fruits were sorted to ensure uniformity. The average mass of a single fruit was 22.1 ± 3.5 g, the average equatorial diameter was 36.5 ± 2.8 mm, and the extract content measured as °Brix was 10.2 ± 0.5 °Brix. Only fruits at commercial ripeness, undamaged and free from visible defects, were selected for analysis.

Strawberries are highly perishable fruits, and their rapid postharvest deterioration requires the use of preservation methods to maintain quality during distribution. In commercial practice, fruits typically undergo several logistics stages—including harvesting, sorting, packaging, transport, and retail display—which often last several days, even under refrigeration. For this reason, a 7-day storage period at 2 ± 1 °C was selected, as it reflects a realistic short-term distribution scenario for fresh strawberries. This duration captures the key physicochemical and microbiological changes relevant to commercial handling and allows for a reliable evaluation of the effectiveness of ozone fumigation.

### 3.2. Ozone Treatment

Strawberry fruits were exposed to gaseous ozone in a fumigation chamber with internal dimensions of 0.8 m (length), 0.5 m (width), and 0.4 m (height). The chamber was connected via a hose to a Korona L5 ozone generator (Laboratorium Naukowo-Wdrożeniowe “Korona”, Piotrków Trybunalski, Poland).

The ozone concentration inside the chamber was continuously monitored and recorded using a UV-based ozone analyzer (Model 106-M, 2B Technologies, Broomfield, CO, USA). The instrument features a measurement uncertainty of ±2% of the reading and a measurement range of 0–1000 ppm. Prior to each experimental session, the analyzer underwent performance verification using its built-in diagnostic and zero-check functions. The device operated under factory calibration, as no external recalibration was performed during the study period.

Environmental conditions inside the ozone chamber were controlled throughout the fumigation process. The chamber temperature during ozone exposure was maintained at 20 ± 1 °C, while the relative humidity inside the chamber was approximately 45%, reflecting the ambient laboratory conditions at the time of treatment. Each experimental variant was performed in three independent replicates. Ozone fumigation was applied once, immediately after harvest. Following treatment, the fruits were placed into 0.5 kg containers and stored at 2 ± 1 °C and 90% relative humidity for 7 days.

In the laboratory experiment, gaseous ozone fumigation was applied using the following dose–time combinations:

- 0 ppm for 0 min (control),
- 10 ppm for 10 min,
- 10 ppm for 20 min,
- 50 ppm for 10 min,
- 50 ppm for 20 min.

During ozone fumigation, strawberries were arranged in a single layer on a perforated stainless-steel tray placed in the center of the fumigation chamber. This layout ensured uniform exposure of all fruits to the gaseous ozone stream and prevented overlapping or physical contact between fruits, which could interfere with gas distribution. For each fumigation variant, approximately 0.50 kg of fruit was placed in the chamber, corresponding to one experimental replicate. The total fruit mass inside the chamber never exceeded 0.50 ± 0.02 kg to maintain consistent surface to volume ratios across treatments.

The selected ozone concentrations (10 and 50 ppm) and exposure times (10 and 20 min) were chosen based on: (1) previously published studies demonstrating that ozone doses within this range influence microbial reduction and physiological responses in strawberries and other berry fruits; (2) preliminary pilot trials conducted in our laboratory, which confirmed that these doses did not cause visible tissue damage and ensured stable, uniform ozone dispersion in the fumigation chamber;

### 3.3. Moisture Content Measurement

Moisture content in fresh strawberry fruits was measured on days 1, 4, and 7 of storage using the oven-drying gravimetric method. The samples (sliced fruits) were dried in a laboratory drying oven SLW 115 SMART (POL-EKO Aparatura Sp.j., Wodzisław Śląski, Poland) at 105 °C until a constant dry mass was obtained.

### 3.4. Mechanical Properties—Skin and Flesh Penetration Test

The mechanical resistance of strawberry skin and flesh to puncture damage was evaluated using a penetration test. Measurements were performed on fresh fruit samples of uniform size and maturity characteristic of the cultivar, subjected to different ozone doses and stored for 7 days. For each experimental variant, 36 replicates were analyzed.

The penetration tests were carried out using a universal testing machine Zwick/Roell Z010 (ZwickRoell GmbH & Co. KG, Ulm, Germany) under the following conditions: initial force at puncture F = 0.5 N, crosshead speed during the penetration test 0.5 mm·s^−1^, and puncture probe diameter 3 mm.

### 3.5. Antioxidant Potential, Vitamin C

The determination of the ascorbic acid content in strawberry fruits was carried out in accordance with the PN-A-04019:1998 standard [89]. Antioxidant activity: DPPH test was determined according to the methodology described by Djordjević et al. [90], the ABTS test was determined according to the methodology described by Jakobek et al. [91]. In turn, the FRAP test was determined according to the methodology described by Chiabrando and Giacalone [92]. All analyses were performed in triplicate.

### 3.6. Profile of Phenolic Compounds

Strawberry fruits collected at harvest maturity from the tested experimental variants were analyzed to determine the effect of the tested factors on the phenolic compound profile. The phenolic compound profile in strawberry fruits was determined using the UPLC-PDA-MS/MS method according to the protocol proposed by Kapusta et al. [93].

### 3.7. Microbial Load

Strawberry fruits were subjected to microbiological analysis on the first and seventh day after plant ozonation. The number of aerobic mesophilic bacteria and yeast and mold counts were determined according to the methodology described by Matłok et al. [94]. Microbiological assessments were performed starting on Day 1, which represents the first sampling point after ozone fumigation; therefore, Day 0 measurements were not included in the experimental design. The analyses were intended to capture the early (Day 1) and advanced (Day 7) stages of microbial development during storage.

### 3.8. Measurements of Gas Concentrations

In the climatic chambers where strawberries from each experimental treatment were stored, the concentrations of CO_2_ (%) and C_2_H_4_ (ppm) in the internal atmosphere were monitored. Each chamber had internal dimensions of 50 × 30 × 20 cm (length × width × height), corresponding to an internal volume of approximately 0.03 m^3^. Gas sampling was performed by positioning the sampling probe of the F-950 analyzer at a fixed location approximately 5 cm above the fruit surface in the central part of the chamber. This placement ensured representative measurements of the atmosphere surrounding the strawberries, minimizing potential bias from wall zones or areas with restricted air circulation.

For each experimental variant, twenty measurements were taken. Gas concentrations were determined using an F-950 analyzer (STEP Systems GmbH, Nuremberg, Germany). During measurements, the airflow drawn from the chambers was maintained at 33 mL·min^−1^, in accordance with the manufacturer’s recommendations. All measurements were conducted 60 min after the completion of the ozonation procedure to allow the chamber atmosphere to stabilize before analysis.

### 3.9. Statistical Analysis

The obtained results were analyzed using STATISTICA 13.1 software (StatSoft, Palo Alto, CA, USA). A one-way analysis of variance (ANOVA) was performed separately for each storage day to evaluate the effect of ozone concentration and exposure time on the analyzed parameters. Statistically significant differences compared with the control were indicated in the figures with asterisks (*). Data are presented as mean values ± standard deviation (SD).

## 4. Conclusions

The conducted study demonstrated that the application of gaseous ozone is an effective method for improving the quality and extending the short-term storage life of strawberry fruits. Ozone treatment significantly reduced the intensity of metabolic processes, as confirmed by lower CO_2_ and ethylene concentrations during storage. Moreover, ozonation contributed to preserving the nutritional value of the fruit by maintaining higher vitamin C content and enhanced antioxidant activity. Importantly, fumigation with ozone did not negatively affect the mechanical properties of the strawberries, indicating that the applied doses were gentle on fruit tissue structure. The strongest antimicrobial effect was observed at the higher ozone concentration, which resulted in a pronounced inhibition of mesophilic bacteria, yeasts, and molds during refrigerated storage.

However, the present study did not include an assessment of possible ozone-derived by-products nor an evaluation of worker exposure to ozone, which are essential aspects of safety before this technology can be implemented on an industrial scale. Therefore, future research should incorporate measurements of residual oxidants, ensure compliance with occupational exposure limits, and develop process designs that minimize human contact with ozone. Another limitation of the study is the lack of sensory (organoleptic) evaluation. Although no negative effects on the mechanical properties of the fruit were detected, the potential influence of ozone on flavor, aroma, and overall consumer acceptance remains unknown and requires further investigation. It should also be emphasized that although ozone decomposes to oxygen and does not leave permanent residues, it is still classified as a biocide and cannot be described as “environmentally friendly” in a strict regulatory sense. Its potential advantages lie primarily in on-site generation and the absence of persistent residues compared with many conventional chemical disinfectants.

In conclusion, the results indicate that gaseous ozone represents a promising alternative postharvest treatment for strawberries. Nevertheless, implementation of this technology on a larger scale requires further research addressing safety aspects, sensory quality, optimal dose–time combinations, and operational constraints associated with the use of ozone in commercial storage facilities.

## Figures and Tables

**Table 1:** Total polyphenol content and phenolic compound profile in strawberry fruits during storage [mg·kg^−1^].

No.	Compound	Day	Control	10 ppm 10 min	10 ppm 20 min	50 ppm 10 min	50 ppm 20 min
1	3-O-glucoside of Pelargonidin	1	233.25	145.96	205.99	197.05	233.74
		4	205.77	237.87	189.13	137.43	184.02
		7	182.65	303.92	228.10	167.82	177.84
2	3-O-rutinoside of Pelargonidin	1	7.29	3.78	5.75	5.12	6.57
		4	6.26	5.43	5.01	4.29	3.68
		7	6.96	6.29	5.42	6.95	4.76
3	3-O-glucoside of Pelargonidin–malonyl derivative	1	83.29	40.34	54.95	65.08	69.00
		4	62.61	62.00	60.32	39.59	68.50
		7	68.03	67.21	41.40	62.59	71.85
4	3-O-glucoside of p-coumaric acid	1	4.34	3.93	4.35	3.00	5.11
		4	3.89	6.30	3.64	2.43	2.97
		7	3.24	8.03	6.07	3.08	3.20
5	Unidentified derivative of ferulic acid	1	2.55	1.56	2.11	2.14	2.21
		4	2.19	2.31	2.02	1.65	2.01
		7	1.78	3.00	2.20	1.85	1.84
6	3-O-sophoroside of Kaempferol	1	0.99	0.54	0.67	0.81	0.81
		4	0.78	0.76	0.81	0.54	0.87
		7	0.77	0.89	0.53	0.70	0.95
7	3-O-glucuronide of Quercetin	1	2.69	2.86	3.18	3.12	2.39
		4	3.13	3.03	2.42	3.19	2.91
		7	3.00	4.56	3.56	4.61	2.82
8	3-O-glucuronide of Kaempferol	1	0.95	1.06	1.35	1.05	1.27
		4	1.15	1.28	0.89	0.98	0.74
		7	0.85	1.91	1.59	0.95	0.76
9	3-O-glucoside of Kaempferol–acetyl derivative	1	1.07	0.64	0.96	0.87	1.10
		4	0.96	1.03	0.74	0.63	0.76
		7	0.76	1.16	1.01	0.73	0.75
	**Total**	1	336.42	200.67 *	279.31 *	278.24 *	322.20
		4	286.74	319.98 *	264.98	190.73 *	266.46
		7	268.04	396.97 *	289.88	249.28	264.77

Asterisks (*) indicate significant differences between ozone-treated samples and the control within the same storage day (*p* ≤ 0.05). Values without asterisks do not differ significantly from the control.

## Data Availability

The original contributions presented in this study are included in the article. Further inquiries can be directed to the corresponding authors.
